# Supplementary material for: Embryonic Thermal Manipulation Affects the Antioxidant Response to Post-Hatch Thermal Exposure in Broiler Chickens
Source: Animals (Basel). 2020 Jan 13;10(1):126. doi: 10.3390/ani10010126 (PMC7022970; doi:10.3390/ani10010126)
Supplement: Supplementary file 1 [file animals-10-00126-s001.pdf]

Supplementary Materials

Embryonic Thermal Manipulation Affects the Antioxidant Response to Post-Hatch Thermal Exposure in Broiler Chickens

Khaled M. M. Saleh [1], Amneh H. Tarkhan [1] and Mohammad Borhan Al-Zghoul [2,*]

Table 1. effects of heat stress on the hepatic mRNA levels of certain antioxidant enzymes in broiler chickens subjected to embryonic TM.

Liver	Control		TM	
**NOX4**	**TN**	**CHS**	**TN**	**CHS**
**Day 0**	1.00 ± 0.20 ^{aw}	1.03 ± 0.20 ^{aw}	1.09 ± 0.10 ^{ax}	1.13 ± 0.10 ^{aw}
**Day 1**	1.03 ± 0.17 ^{aw}	2.21 ± 0.37 ^{bx}	1.13 ± 0.13 ^{ax}	2.39 ± 0.35 ^{bx}
**Day 3**	1.01 ± 0.14 ^{aw}	6.09 ± 1.26 ^{by}	1.03 ± 0.10 ^{ax}	1.78 ± 0.46 ^{cx}
**Day 5**	1.02 ± 0.10 ^{aw}	5.04 ± 2.37 ^{by}	1.08 ± 0.10 ^{ax}	1.4 ± 0.14 ^{cx}
**Day 7**	1.01 ± 0.20 ^{aw}	8.63 ± 4.41 ^{by}	1.15 ± 0.10 ^{ax}	1.51 ± 0.31 ^{cx}
**SOD**	**control**	**TM**	**control**	**TM**
**Day 0**	1.00 ± 0.12 ^{aw}	1.05 ± 0.42 ^{aw}	0.64 ± 0.13 ^{ax}	0.64 ± 0.13 ^{aw}
**Day 1**	1.11 ± 0.32 ^{aw}	1.90 ± 0.17 ^{bx}	0.67 ± 0.11 ^{ax}	1.68 ± 0.79 ^{cx}
**Day 3**	1.11 ± 0.42 ^{aw}	1.76 ± 0.39 ^{bx}	0.54 ± 0.13 ^{ax}	0.17 ± 0.07 ^{cy}
**Day 5**	1.09 ± 0.22 ^{aw}	1.86 ± 0.08 ^{bx}	0.69 ± 0.14 ^{ax}	0.91 ± 0.15 ^{aw}
**Day 7**	0.97 ± 0.42 ^{aw}	3.5 ± 1.50 ^{bx}	0.64 ± 0.17 ^{ax}	0.8 ± 0.12 ^{aw}
**Catalase**	**TN**	**CHS**	**TN**	**CHS**
**Day 0**	1.00 ± 0.23 ^{aw}	1.03 ± 0.33 ^{aw}	0.47 ± 0.23 ^{bw}	0.37 ± 0.23 ^{bw}
**Day 1**	1.13 ± 0.33 ^{aw}	2.54 ± 0.29 ^{bx}	0.39 ± 0.19 ^{cw}	2.23 ± 0.86 ^{ax}
**Day 3**	0.93 ± 0.22 ^{aw}	1.65 ± 0.37 ^{by}	0.47 ± 0.19 ^{cw}	0.91 ± 0.14 ^{aw}
**Day 5**	1.03 ± 0.14 ^{aw}	3.98 ± 0.14 ^{bx}	0.42 ± 0.23 ^{cw}	0.75 ± 0.25 ^{cw}
**Day 7**	1.13 ± 0.29 ^{aw}	2.70 ± 1.00 ^{bx}	0.51 ± 0.23 ^{cw}	0.94 ± 0.08 ^{aw}

Control: control group, TM: thermal manipulated group, TN: Thermoneutral, CHS: chronic heat, NOX4: NADPH oxidase 4 SOD: superoxide dismutase 2.

$^{a-c}$ within same row, mean ±SD with different superscript differ significantly (p-value < 0.05)

$^{w-z}$ within same treatment column, mean ±SD with different superscript differ significantly (p-value < 0.05)

Table S2: effects of heat stress on the splenic mRNA levels of certain antioxidant enzymes in broiler chickens subjected to embryonic TM.

Spleen	Control		TM	
**NOX4**	**TN**	**CHS**	**TN**	**CHS**
**Day 0**	1.00 ± 0.23 aw	1.13 ± 0.38 bw	2.01 ± 1.01 aw	2.21 ± 1.51 aw
**Day 1**	1.03 ± 0.27 aw	0.62 ± 0.11 bw	1.99± 1.05 aw	0.47 ± 0.13 bx
**Day 3**	1.03 ± 0.33 aw	0.45 ± 0.09 aw	1.75 ± 1.51 aw	0.56 ± 0.06 ax
**Day 5**	1.03 ± 0.23 aw	2.9 ± 1.05 bx	2.03 ± 1.11 aw	0.61 ± 0.05 cx
**Day 7**	1.17 ± 0.23 aw	1.09 ± 0.41 ay	2.31 ± 1.21 aw	0.39 ± 0.14 bx
**SOD**	**TN**	**CHS**	**control**	**TM**
**Day 0**	1.00 ± 0.23 aw	1.03 ± 0.46 aw	0.86 ± 0.33 aw	0.86 ± 0.65 aw
**Day 1**	1.17 ± 0.23 aw	1.52 ± 1.04 ax	0.89 ± 0.55 aw	1.77 ± 0.93 ax
**Day 3**	1.03 ± 0.27 aw	2.39 ± 0.24 cy	0.75 ± 0.45 aw	1.54 ± 0.2 bx
**Day 5**	1.03 ± 0.33 aw	2.98 ± 0.84 by	0.86 ± 0.35 aw	3.53 ± 0.85 by
**Day 7**	1.13 ± 0.25 aw	4.15 ± 0.96 bz	0.90 ± 0.55 aw	1.42 ± 0.39 ax
**Catalase**	**TN**	**CHS**	**TN**	**CHS**
**Day 0**	1.00 ± 0.27 aw	1.04 ± 0.45 aw	1.45 ± 1.13 aw	1.45 ± 1.13 aw
**Day 1**	1.03 ± 0.33 aw	6.4 ± 1.85 bx	1.35 ± 1.33 aw	1.59 ± 1.11 aw
**Day 3**	1.13± 0.23 aw	2.09 ± 0.21 ay	1.45 ± 0.97 aw	5.71 ± 1.39 ax
**Day 5**	1.07 ± 0.27 aw	6.81 ± 0.22 ax	1.55 ± 0.87 aw	5.58 ± 1.16 ax
**Day 7**	1.09 ± 0.21 aw	15.54 ± 6.85 az	1.15 ± 1.23 aw	2.21 ± 1.65 aw

Control: control group, TM: thermal manipulated group, TN: Thermoneutral, CHS: chronic heat, NOX4: NADPH oxidase 4 SOD: superoxide dismutase 2.

a-c within same row, mean ±SD with different superscript differ significantly (p-value < 0.05)

w-z within same treatment column, mean ±SD with different superscript differ significantly (p-value < 0.05)
